# A new automatic algorithm for quantification of myocardial infarction imaged by late gadolinium enhancement cardiovascular magnetic resonance: experimental validation and comparison to expert delineations in multi-center, multi-vendor patient data

**DOI:** 10.1186/s12968-016-0242-5

**Published:** 2016-05-04

**Authors:** Henrik Engblom, Jane Tufvesson, Robert Jablonowski, Marcus Carlsson, Anthony H. Aletras, Pavel Hoffmann, Alexis Jacquier, Frank Kober, Bernhard Metzler, David Erlinge, Dan Atar, Håkan Arheden, Einar Heiberg

**Affiliations:** Department of Clinical Sciences Lund, Clinical Physiology, Lund University, Skåne University Hospital, Lund, Sweden; Department of Biomedical Engineering, Faculty of Engineering, Lund University, Lund, Sweden; Laboratory of Medical Informatics, School of Medicine, Aristotle University of Thessaloniki, Thessaloniki, Greece; Section for Interventional Cardiology, Department of Cardiology, Division of Cardiovascular and Pulmonary Diseases, Oslo University Hospital, Ullevaal, Oslo, Norway; Aix-Marseille University, UMR 7339 CRMBM, Marseille, France; Department of Radiology, La Timone University Hospital, Marseille, France; Department of Cardiology, Medical University of Innsbruck, Innsbruck, Austria; Department of Cardiology, Lund University, Lund, Sweden; Department of Cardiology B, Oslo University Hospital Ullevål and Faculty of Medicine, University of Oslo, Oslo, Norway; Department of Clinical Physiology and Nuclear Medicine, Skåne University Hospital, SE-221 85 Lund, Sweden

**Keywords:** LGE CMR, Automatic quantification algorithm, Expectation maximization, Experimental validation, Multi-center patient data

## Abstract

**Background:**

Late gadolinium enhancement (LGE) cardiovascular magnetic resonance (CMR) using magnitude inversion recovery (IR) or phase sensitive inversion recovery (PSIR) has become clinical standard for assessment of myocardial infarction (MI). However, there is no clinical standard for quantification of MI even though multiple methods have been proposed. Simple thresholds have yielded varying results and advanced algorithms have only been validated in single center studies. Therefore, the aim of this study was to develop an automatic algorithm for MI quantification in IR and PSIR LGE images and to validate the new algorithm experimentally and compare it to expert delineations in multi-center, multi-vendor patient data.

**Methods:**

The new automatic algorithm, EWA (*E*xpectation Maximization, *w*eighted intensity, *a* priori information), was implemented using an intensity threshold by Expectation Maximization (EM) and a weighted summation to account for partial volume effects.

The EWA algorithm was validated in-vivo against triphenyltetrazolium-chloride (TTC) staining (*n* = 7 pigs with paired IR and PSIR images) and against ex-vivo high resolution T1-weighted images (*n* = 23 IR and *n* = 13 PSIR images). The EWA algorithm was also compared to expert delineation in 124 patients from multi-center, multi-vendor clinical trials 2–6 days following first time ST-elevation myocardial infarction (STEMI) treated with percutaneous coronary intervention (PCI) (*n* = 124 IR and *n* = 49 PSIR images).

**Results:**

Infarct size by the EWA algorithm in vivo in pigs showed a bias to ex-vivo TTC of −1 ± 4%LVM (*R* = 0.84) in IR and −2 ± 3%LVM (*R* = 0.92) in PSIR images and a bias to ex-vivo T1-weighted images of 0 ± 4%LVM (*R* = 0.94) in IR and 0 ± 5%LVM (*R* = 0.79) in PSIR images. In multi-center patient studies, infarct size by the EWA algorithm showed a bias to expert delineation of −2 ± 6 %LVM (*R* = 0.81) in IR images (*n* = 124) and 0 ± 5%LVM (*R* = 0.89) in PSIR images (*n* = 49).

**Conclusions:**

The EWA algorithm was validated experimentally and in patient data with a low bias in both IR and PSIR LGE images. Thus, the use of EM and a weighted intensity as in the EWA algorithm, may serve as a clinical standard for the quantification of myocardial infarction in LGE CMR images.

**Clinical trial registration:**

CHILL-MI: NCT01379261. MITOCARE: NCT01374321.

## Background

Late gadolinium enhancement (LGE) cardiovascular magnetic resonance (CMR) is considered the reference standard for the assessment of myocardial infarction (MI) [1, 2]. Visualization of MI by use of gadolinium enhancement has evolved from T1-weighted imaging in 1984 [3] to current use of LGE magnitude inversion recovery (IR) [4] and phase sensitive inversion recovery (PSIR) sequences [5] as clinical standard [6].

However, there is no clinical standard for quantification of MI in LGE images even though multiple methods have been proposed [6]. Manual delineation or visual grading of MI is often used clinically but has the disadvantage of being subjective, and therefore threshold techniques have been proposed based on different numbers of standard deviations (SD) from remote myocardium or based on the full width half maximum (FWHM) intensity threshold [7–9]. These different approaches yield highly variable results [10]. Recently Stirrat et al. [11] showed a difference between infarct size derived from IR and PSIR LGE images for threshold methods of SD from remote and FWHM. More advanced methods for MI quantification have been implemented and validated as the FACT algorithm by Hsu et al. [12, 13] and the weighted algorithm by Heiberg et al. [14]. Both algorithms involve regional analysis of the infarcted myocardium to include microvascular obstruction (MVO) and exclude artifacts. However, the FACT algorithm [12] was developed and validated for PSIR images with surface coil intensity correction and based on a FWHM threshold, whereas the weighted algorithm [14] was developed and validated for magnitude IR images. Heiberg et al. [14] used a weighted approach to account for partial volume effects, which was shown to decrease variability compared to the use of pure signal intensity thresholds. The algorithm was, however, based on a SD threshold from remote, and the weighted approach was not applied in ex-vivo high resolution T1-weighted images. Using a threshold by Expectation Maximization (EM) [15] has been shown superior to FWHM and SD from remote for quantification of myocardium at risk in T2-weighted images [16], and the EM-algorithm has also been implemented for MI quantification in LGE images [17]. The EM-algorithm has previously not been combined with a weighted approach and, to the best of our knowledge, no algorithms have been developed for MI quantification in both IR and PSIR LGE images and applied in multi-center, multi-vendor patient studies.

Therefore, the aim of this study was 1) to develop a new automatic algorithm for MI quantification by combining intensity threshold by Expectation Maximization (EM) with a weighted approach to account for partial volume effects, 2) to validate the automatic algorithm experimentally for IR and PSIR LGE images against ex-vivo TTC stained slices and ex-vivo high resolution T1-weighted images, and 3) compare the automatic algorithm in multi-center, multi-vendor patient data to consensus expert delineations as reference as well as compare the applicability of the new automatic algorithm to previously suggested methods for infarct quantification in both IR and PSIR LGE images.

## Methods

### Study population

#### Experimental studies

Pigs with induced myocardial infarction were included from three previous studies, one mechanistic study of myocardial infarction (*n* = 15) [18], one cardioprotection study (*n* = 15) [19] and controls from one cardioprotection study previously used for validating the original weighted algorithm for infarct quantification (*n* = 8) [14]. All three animal studies conformed to the Guide for the Care and Use of Laboratory Animals United States National Institutes of Health (NIH Publication No.85-23, revised 1996) and were approved by the Regional Ethics Committee. The experimental protocols for each of the studies have been previously published [14, 18, 19]. In short, all pigs were subjected to 40 min occlusion with a balloon placed after the first or the second diagonal branch of the left anterior descending artery (LAD). Myocardial infarction was imaged after four hours [14, 19], six hours [18] or seven days [18] of reperfusion with either in-vivo 3D IR LGE (*n* = 23), in-vivo 2D PSIR LGE (*n* = 13) and/or ex-vivo high resolution (0.5 × 0.5 × 0.5 mm) T1-weighted images (*n* = 38). CMR imaging was performed on a 1.5 T Philips scanner (Philips Healthcare, Best, The Netherlands). In-vivo LGE images were acquired approximately 20 min after injection of gadolinium-based contrast agent (typical resolution 1.5 × 1.5 × 8.0 mm, no slice gap). Ex-vivo high resolution (0.5 × 0.5 × 0.5 mm) T1-weighted images were acquired covering the entire left ventricle (LV) with the explanted hearts placed in plastic containers and the ventricles filled with balloons containing deuterated water. For ex-vivo imaging, a gadolinium-based contrast agent was administered 15 min prior to administration of a potassium chloride bolus. Seven pigs with MI were imaged, both in-vivo and ex-vivo, after seven days of reperfusion and following ex-vivo imaging, hearts were sliced into five mm slices and incubated in triphenyltetrazolium-chloride (TTC) for five minutes. The slices were subsequently photographed on both apical and basal sides for infarct analysis.

#### Patient population

**Fig. 1:**
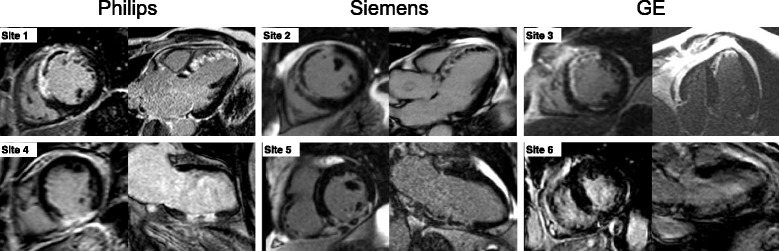
Examples of LGE images from different sites using different vendors. Representative images (a mid-ventricular short-axis image and a long-axis image) from six different sites using either a Philips, Siemens or GE scanner. The upper panel shows images from three patients with anteroseptal infarction due to LAD occlusion. The lower panel shows images from three patients with inferior infarction due to RCA occlusion

Patients with first time ST-elevation myocardial infarction (STEMI) treated with percutaneous coronary intervention (PCI) were included from the recently published clinical cardioprotection trials CHILL-MI [20] (*n* = 58) and MITOCARE [21] (*n* = 66). Patients underwent CMR imaging within 2–6 days following acute MI treated with PCI. Inclusion and exclusion criteria for each of the clinical trials have been previously published [20, 22]. In short, all patients had clinical signs of acute MI defined as clinical symptoms and ECG signs consistent with ST-elevation infarction or new onset of left bundle branch block (LBBB), were ≥ 18 years old and had symptom duration of less than 6 h. Patients with a history of previous myocardial infarction or history of coronary revascularization were excluded. Both studies [20, 21] from which patients were recruited were approved by the institutional review boards/Ethics Committees, and all patients provided written informed consent. No specific ethics approval or informed consent was needed for the development of the new algorithm in the current study. All CMR examinations were performed on 1.5 T scanners from Philips (Philips Healthcare, Best, The Netherlands), Siemens (Siemens Healthcare, Erlangen, Germany) or GE (GE Healthcare, Waukesha, WI, USA). For infarct assessment, LGE images covering the entire LV were acquired approximately 15 min after injection of the gadolinium-based contrast agent. The LGE-images were acquired using a magnitude inversion-recovery (IR) or phase sensitive inversion recovery (PSIR) gradient-recalled echo sequence with a typical resolution of 1.5 × 1.5 × 8.0 mm, no slice gap [4]. Inversion time was manually adjusted to null the signal of viable myocardium. Surface coil intensity correction was not mandatory across vendors and sites. This study included patients who had undergone CMR with LGE magnitude IR images (*n* = 75) or paired LGE magnitude and phase sensitive IR images (*n* = 49). Image quality was assessed as [1] poor, [2] acceptable or [3] good, where acceptable and good images were considered for this study. Examples of representative LGE images from different sites using different vendors are shown in Fig. 1.

### Image analysis

All images were analyzed using the software Segment (http://segment.heiberg.se) [23]. For all analysis (clinical and experimental) endocardial and epicardial borders were traced manually with exclusion of the papillary muscles. Infarct size was expressed as % of left ventricular mass (LVM).

#### Patient population

In LGE images, infarct expert delineation was performed using the weighted method previously validated and published for IR imaging [14], with manual checking to correct for obvious artifacts and failure of the computer algorithm to correctly identify infarction due to i.e. high noise levels. Hypointense regions within the region of gadolinium enhancement were considered to be MVO [24] and were included in the analysis as 100 % infarction. Delineation of each data set was performed by one of three primary observers (HE, MC and HA with 14, 15 and 20 years of experience, respectively) in a core lab setting (Imacor AB, Lund Sweden) with a quality control of the delineations by a second observer for each case. Different opinions for the delineations were resolved in consensus between all three observers when necessary. In a subset of 17 patients a second-observer delineation was performed using the same endocardial and epicardial borders to evaluate inter-observer variability of the expert delineation (MC vs. HE).

#### Experimental studies

For the experimental in-vivo data, LGE images were delineated with the same method as for the patients by one observer (RJ with 5 years of CMR experience). In the high resolution T1-weighted images, infarct delineation was performed using a threshold of 8SD from remote [14], with manual corrections where needed (RJ or HE). Hypointense regions were considered to be MVO and included in the infarct delineation. Infarct quantification in the TTC images was performed manually by one observer, delineating the non TTC-stained parts of the myocardium as infarction (RJ).

### Automatic quantification of MI

**Fig. 2:**
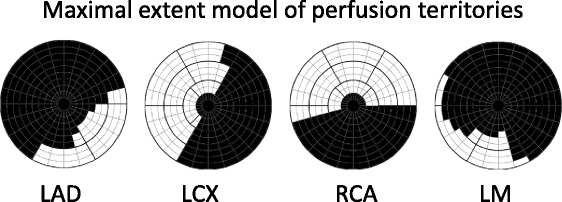
Maximal extent model of perfusion territories. Bulls-eye representation of maximal extent model of the perfusion territories of left anterior descending artery (LAD), left circumflex artery (LCx), right coronary artery (RCA), and left main artery (LM). Models for LAD, LCX and RCA were defined in consensus by three experienced observers in an extended 17- segment AHA model, and models for LM were defined from the models of LAD, LCX and RCA. The 17-segment model is extended to three slices in each of the basal, mid-ventricular and apical zones and 24 sectors in each slice. Black sectors are included in the maximal extent model. The septal part of the left ventricle was represented in the left of the bulls-eye plot, the lateral part in the right, anterior part in the top, inferior part in the bottom, the apical slices in the center and the basal slices in the outer part of the bulls-eye plot

The automatic algorithm for MI quantification was implemented and incorporated in the freely available software Segment [23]. The new automatic algorithm, EWA, is based on three major principles: *E*xpectation Maximization for intensity classification, *w*eighted summation of infarct size to account for partial volume effects according to pixel intensity and a priori information utilized for pre and post processing. A maximal extent model of the perfusion territories [16] was used as a priori information and was defined by consensus between three experienced observers for each culprit artery (Fig. 2). The user supplies the EWA algorithm with information on culprit artery and indicates the rotation of the left ventricle by the inferior and anterior right ventricular insertion points. The EWA algorithm consists of six steps:

1. Surface coil intensity correction
2. Classification of myocardial intensities by means of an EM-algorithm
3. Segmentation of infarct region by means of a level set method
4. Inclusion of microvascular obstruction
5. Post processing to exclude artifacts
6. Calculation of the infarct size by weighting the pixels based on their intensity.

In *step 1*, surface coil intensity correction was applied as a second order linear correction to be able to account for intensity gradient proportional to the squared coil distance and was based on the intensities in the blood pool and remote myocardium. The remote myocardium was defined by using the maximal extent model of the culprit artery [16].

In *step 2*, an EM-algorithm [15] was used to classify myocardial pixel intensities as representative of normal myocardium or infarct. A constrained EM-algorithm iteratively refined an initial classification to find the maximum likelihood estimate of the mean and standard deviation for the Gaussian intensity distributions of normal myocardium and MI. The initial classification was defined as a small MI of 10 % by a pure threshold at the 90th percentile of the intensity histogram. The constraint was set to force pixel intensities below the 5th percentile classified as normal myocardium and pixel intensities above the 95th percentile classified as MI. The constraint was set in order to increase the stability of the EM-algorithm and ensure separation between the two estimated Gaussian distributions representative of normal myocardium and MI. The optimal intensity threshold was defined from the mean and standard deviation estimated by the EM-algorithm as the intensity for which there was an equal probability of being representative of normal myocardium and MI.

In *step 3*, the MI region was segmented using a fast level set method [25] in which the main driving force was what pixels to include or exclude based on intensity with parameters for smoothing as previously implemented by Heiberg et al. [14] for quantification of infarction. The seed points for this level set algorithm were all pixels above the calculated threshold in step 2. The main driving force in a level set method is called the speed image and should be defined to be positive for pixels to include and negative for pixels to exclude in the segmentation. Therefore, the speed image was set to a linear function with zero at the optimal threshold defined by EM, 1 at the maximal myocardial intensity and subsequently negative values for intensities below the optimal threshold.

In *step 4*, MVO was detected by means of a flood fill algorithm and morphological operations. Microvascular obstruction is characterized by regions of low intensity within the MI and might not have been detected as MI by thresholding. In the EWA algorithm MVO was detected slice by slice as holes in the infarct region by using a flood fill algorithm as suggested by Heiberg et al. [14] in combination with morphological closing as suggested by Hsu et al. [12]. First a flood fill algorithm was used to detect dark pixels as MVO if totally surrounded by pixels segmented as infarct or connected to the endocardial border. Next a morphological closing operation was performed by first applying a dilation operation with a 3-by-3 pixel cross shaped kernel to close small gaps in the infarct segmentation. Then, the flood fill algorithm was reapplied to find any holes arising from the morphological closing before performing the erosion operation.

In *step 5*, post processing of the MI segmentation was performed in two steps: removing pixels classified as MI outside the culprit region and removing small isolated regions classified as MI. By using the same maximal extent model as for surface coil correction, bright regions outside the culprit artery region could be removed from the MI segmentation. Regions segmented as MI which were smaller than 1.5 cm^3^ were removed regardless of location if not comprising more than 1 % of the left ventricular mass or if being the only region of MI.

In *step 6*, the final step, the MI size was calculated by a weighted summation, where each pixel within the MI was weighed according to its intensity to account for partial volume effects. The weight represented the amount of infarcted cells within the pixel and hence in normal myocardium the weight should be 0 and in pixels with the maximal intensity the weight should be set to 1. The weight for each pixel was calculated as a linear function from weight 0 at the mean intensity of the remote myocardium to weight 1 at the 90th percentile of the intensities within the MI.

The maximal extent model of the culprit artery was needed for the intensity correction in step 1 and the first part of the post processing in step 5. However, the maximal extent model could not be applied in experimental studies where the anatomy differs and therefore the algorithm was used without the use of maximal extent model and user input of insertion points and culprit artery in the experimental part of this study.

### Statistical analysis

Experimental validation: Infarct size by the EWA algorithm and infarct size by the expert delineation in in-vivo IR, in-vivo PSIR and ex-vivo high resolution T1-weighted images was compared to infarct size by TTC for myocardial infarction imaged seven days after reperfusion. Infarct size by the EWA algorithm in in-vivo IR, in-vivo PSIR and ex-vivo high resolution T1-weighted images was compared to infarct size by expert delineation in ex-vivo high resolution T1-weighted images regardless of timing of imaging. Comparisons were performed using Bland-Altman bias (mean ± standard deviation) and linear regression analysis (correlation coefficient).

Applicability in patient data: Infarct size by the EWA algorithm was compared to infarct size by expert delineation using Bland-Altman bias (mean ± standard deviation) and linear regression analysis (correlation coefficient). Performance of the EWA algorithm was compared to the original weighted algorithm by Heiberg et al. [14], and the thresholds of EM, 2, 3 and 5SD from remote, FWHM from minimum intensity as implemented by Amado et al. [8], FWHM from remote intensity as implemented by Hsu et al. [12] and Otsu's threshold [26]. Comparison was performed in paired IR and PSIR LGE images using bias and linear regression analysis with expert delineation as reference. Regional agreement with expert delineation was evaluated using Dice Similarity Coefficient (DSC) [27] for both the full extent of the infarct and the core of the MI as represented if no weighting had been used.

## Results

### Experimental studies

**Fig. 3:**
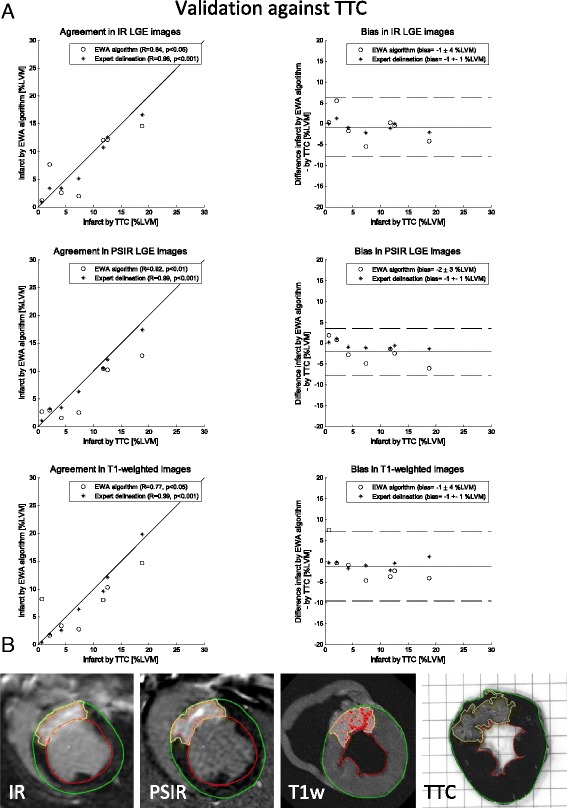
Validation against TTC: **a** Scatter plots (*left column*) and Bland-Altman plots (*right column*) of infarct size expressed as % of left ventricular mass (%LVM) for the EWA algorithm against infarct size by triphenyltetrazolium-chloride (TTC) in pigs with myocardial infarction imaged after seven days (*n* = 7) with in-vivo magnitude inversion recovery LGE images (IR, *top row*), in-vivo phase sensitive inversion recovery LGE images (PSIR, middle row) and ex-vivo high resolution T1-weighted images (T1w, *bottom row*). Left column: solid line = line of identity; dashed line = regression line. Right column: solid line = mean bias; dashed line = mean ± two standard deviations. **b** Infarct segmentation by the EWA algorithm in one pig shown in one slice of in-vivo IR LGE, in-vivo PSIR LGE, ex-vivo high resolution T1w and corresponding TTC-stained slice. Infarct segmentation by the EWA algorithm and by manual delineation in TTC images is shown in yellow. For the automatic EWA segmentation the core of the infarct is shown in pink and microvascular obstruction is shown as the red line within the infarct. Endocardium is delineated in red and epicardium in green

**Fig. 4:**
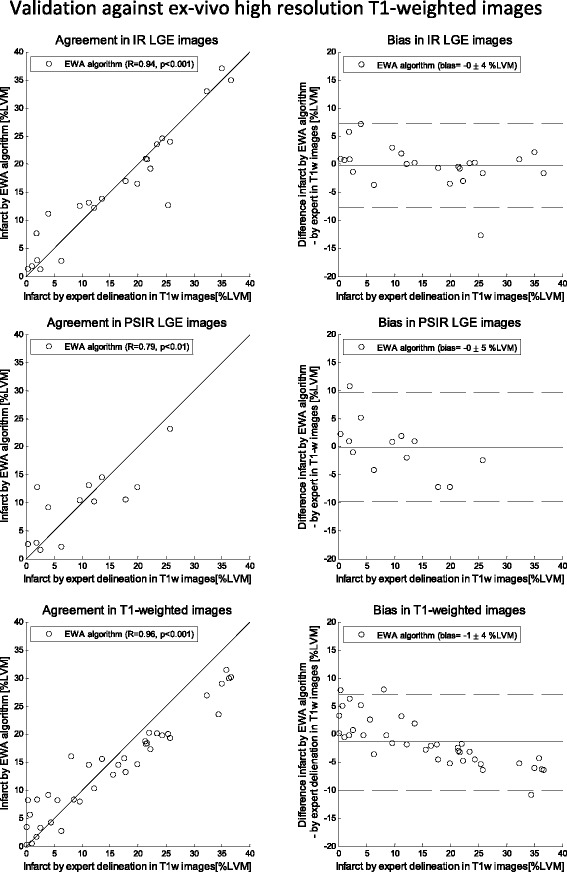
Validation against ex-vivo high resolution T1-weighted images: Scatter plots (*left column*) and Bland-Altman plots (*right column*) of infarct size expressed as % of left ventricular mass (%LVM) for the EWA algorithm against infarct size by expert delineation in ex-vivo high resolution T1-weighted images (T1w). Validation in in-vivo magnitude inversion recovery (IR, top row, *n* = 23 pigs), in-vivo phase sensitive inversion recovery (PSIR, middle row, *n* = 13) and ex-vivo high resolution T1-weighted images (T1w, bottom row, *n* = 38). Left column: solid line = line of identity; dashed line = regression line. Right column: solid line = mean bias; dashed line = mean ± two standard deviations

Infarct size by TTC was 8 ± 6 %LVM (*n* = 7) and infarct size by the EWA algorithm was 7 ± 6 %LVM in in-vivo IR LGE images, 6 ± 5 %LVM in in-vivo PSIR LGE images and 7 ± 5 %LVM in ex-vivo high resolution T1-weighted images (T1w). Fig. 3 shows the agreement and bias for in-vivo IR LGE images, in-vivo PSIR images and ex-vivo high resolution T1-weighted images against TTC. The infarct size by expert delineation in the same seven animals was 8 ± 6 %LVM in IR and PSIR images and 8 ± 7 %LVM in T1w images. For the EWA algorithm the bias to expert delineation in T1w images was 0 ± 4 %LVM (*n* = 23) in IR LGE images, 0 ± 5 %LVM (*n* = 13) in PSIR LGE images and −1 ± 4 %LVM (*n* = 38) in T1w images (Fig. 4).

### Patient population

**Fig. 5:**
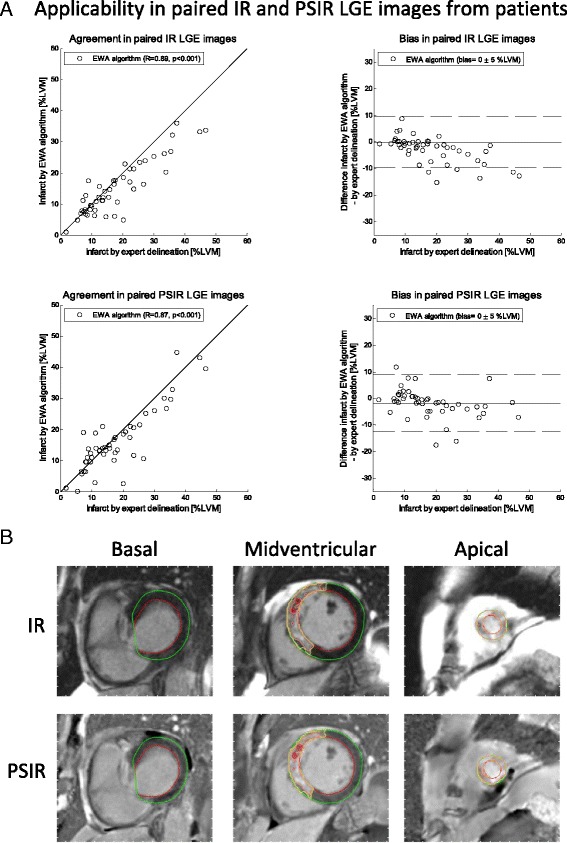
Applicability in paired IR and PSIR LGE images from patients in multi-center, multi-vendor studies: **a** Scatter plots (*left column*) and Bland-Altman plots (*right column*) of infarct size expressed as % of LVM for the EWA algorithm against infarct size by expert delineation in 49 patients from multi-center studies with paired magnitude inversion recovery (IR, *top row*) and phase sensitive inversion recovery LGE images (PSIR, *bottom row*). Left column: solid line = line of identity; dashed line = regression line. Right column: solid line = mean bias; dashed line = mean ± two standard deviations. **b** Typical segmentation by the EWA algorithm in one patient with paired IR (*top row*) and PSIR images (*bottom row*). The automatic EWA segmentation of the infarct is shown in yellow, the core of the infarct is shown in pink and microvascular obstruction is shown as the red line within the infarct. Endocardium is delineated in red and epicardium in green

**Fig. 6:**
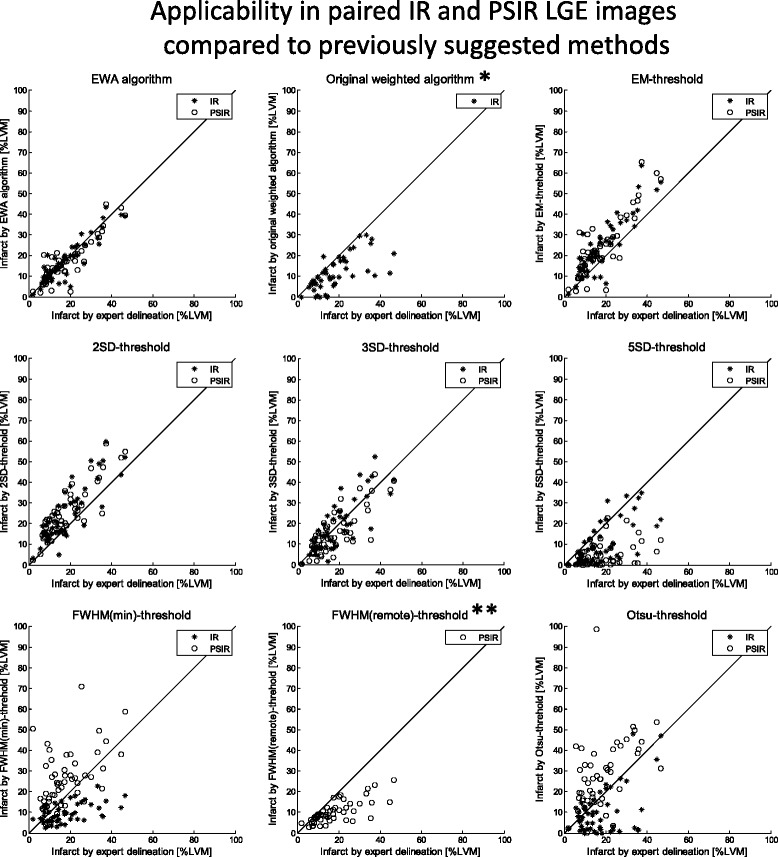
Applicability in paired IR and PSIR LGE images from multi-center patient studies compared to previously suggested methods for MI quantification: Scatter plots of infarct size expressed as % of left ventricular mass (% LVM) against infarct size by expert delineation in 49 patients for the EWA algorithm, the original weighted algorithm [14] and the threshold method of Expectation Maximization (EM) [15] (*top row*), 2SD, 3SD and 5SD from remote (middle row), and FWHM from minimum intensity [8], FWHM from mean intensity in remote [12] and Otsu's threshold [26] (bottom row) in paired magnitude inversion recovery (IR) and phase sensitive inversion recovery (PSIR) LGE images. Solid lines = line of identity. * the original weighted algorithm by Heiberg et al. [14] was developed for IR images and therefore only applied in IR images. ** the FWHM remote threshold was developed for PSIR images as part of the FACT algorithm by Hsu et al. [12] and therefore only applied in PSIR images

Infarct size by the EWA algorithm was 15 ± 8 %LVM in IR images (*n* = 124) with a bias of −2 ± 6 %LVM (*R* = 0.81) compared to the expert delineation. In paired IR and PSIR images (*n* = 49) infarct size by the EWA algorithm was 17 ± 10 %LVM in both IR and PSIR images with a bias of 0 ± 5 %LVM (*R* = 0.89) in IR images and 0 ± 5 %LVM (*R* = 0.87) in PSIR images (Fig. 5). The bias and correlation between expert delineation of infarct size and the EWA algorithm, the original weighted algorithm, thresholds by EM, 2SD, 3SD and 5SD from remote, FWHM from minimum intensity [8], FWHM from mean intensity in remote [12] and Otsu's threshold [26] are summarized in Table 1 and Fig. 6. Inter-observer variability of infarct size by expert delineation was 0 ± 1 %LVM (*R* = 0.99).

**Table 1:** Bias and regional agreement in paired IR and PSIR LGE images from multi-center patient studies

	Magnitude IR LGE				Phase sensitive IR LGE				PSIR vs IR	
	Bias to expert delineation [%LVM]	R-value	DSC full extent	DSC core extent	Bias to expert delineation [%LVM]	R-value	DSC full extent	DSC core extent	Bias [%LVM]	R-value
EWA algorithm	0 ± 5	0.89	0.82 ± 0.14	0.81 ± 0.15	−1 ± 5	0.88	0.82 ± 0.17	0.79 ± 0.15	−1 ± 4	0.91
Original weighted algorithm	−7 ± 8	0.68	0.70 ± 0.32	0.67 ± 0.32	*	*	*	*	*	*
EM threshold	6 ± 7	0.88	-	0.67 ± 0.14	6 ± 8	0.86	-	0.68 ± 0.14	0 ± 6	0.91
2SD threshold	7 ± 7	0.85	-	0.69 ± 0.15	8 ± 6	0.86	-	0.70 ± 0.13	1 ± 5	0.94
3SD threshold	0 ± 7	0.81	-	0.70 ± 0.21	−2 ± 7	0.79	-	0.70 ± 0.19	−2 ± 4	0.94
5SD threshold	−8 ± 8	0.68	-	0.50 ± 0.33	−13 ± 10	0.38	-	0.36 ± 0.31	−4 ± 6	0.81
FWHM (min) threshold	−8 ± 9	0.54	-	0.58 ± 0.20	9 ± 12	0.47	-	0.69 ± 0.17	18 ± 12	0.44
FWHM (remote) threshold	**	**	-	**	−8 ± 7	0.74	-	0.66 ± 0.19	**	**
Otsu threshold	−8 ± 11	0.50	-	0.50 ± 0.32	10 ± 15	0.46	-	0.64 ± 0.20	18 ± 17	0.35

Bias as % of left ventricular mass (%LVM), regression R-value and regional agreement by DSC to expert delineation for the EWA algorithm, the original weighted algorithm [14] and the threshold method of EM, 2SD, 3SD and 5SD from remote, and FWHM from minimum intensity [8], FWHM from mean intensity in remote [12] and Otsu's threshold [26] in paired magnitude inversion recovery (IR) and phase sensitive inversion recovery (PSIR) images (*n* = 49) and bias and regression R-value for PSIR vs IR LGE images. * the original weighted algorithm by Heiberg et al. [14] was developed for IR images and therefore only applied in IR images. ** the FWHM remote threshold was developed for PSIR images as part of the FACT algorithm by Hsu et al. [12] and therefore only applied in PSIR images

## Discussion

This study has presented a new automatic algorithm, the EWA algorithm, for MI quantification based on intensity classification by Expectation Maximization (EM) and weighting each pixel according to its intensity to account for partial volume effects. The EWA algorithm was validated experimentally and compared to expert delineations in multi-center, multi-vendor patient data with a low bias and high regional agreement in both IR and PSIR LGE images. The performance of the EWA algorithm was found superior to several previously described methods for MI quantification and the EWA algorithm was successfully applied to high resolution T1-weighted images, showing the ability of the EWA algorithm to adapt to different image qualities. The superiority of the EWA algorithm compared to other methods such as Otsu and FWHM can be contributed to the combination of the constrained Expectation Maximization for threshold determination and the weighting of the pixels in the infarct quantification.

### Experimental studies

The EWA algorithm was validated against TTC with bias similar to FWHM from minimum intensity as suggested by Amado et al. [8] (4.1 ± 1.1 %LVM, *R* = 0.94) and the FACT algorithm by Hsu et al. [12] (1.9 % LVM, *R* = 0.96). The EWA algorithm was also validated against ex-vivo high resolution T1-weighted images in a larger cohort with bias comparable to the original weighted algorithm by Heiberg et al. [14] (−0.3 ± 1.3 %LVM). LGE in CMR has been shown to overestimate acute MI in comparison to TTC [18, 28, 29] and Jablonowski et al. [18] showed an overestimation by CMR after 6 h of reperfusion which was not seen after seven days of reperfusion. The overestimation in the acute setting was explained by an increased extracellular volume adjacent to the infarct which was not seen after seven days of reperfusion [18]. Thus, in the current study TTC was used as reference in myocardial infarction imaged seven days after reperfusion but ex-vivo high resolution T1-weighted images were used as reference for quantification of acute MI. Ex-vivo high resolution T1-weighted images and inversion recovery LGE are based on the same principle of being proportional to the distribution of the gadolinium based contrast agent in the extracellular volume. Ex-vivo imaging enables high resolution imaging and therefore ex vivo high resolution T1-weighted imaging can be used as reference for in-vivo IR and PSIR LGE in both the acute and chronic setting.

However, neither TTC nor ex-vivo high resolution T1-weighted images can be used for validation in patient studies. In this study, expert delineation was chosen as the reference for MI quantification in patients, performed by using the original weighted algorithm by Heiberg et al. [14] followed by manual corrections and consensus reading. The expert delineation was validated against TTC demonstrating a lower bias (−1 ± 1 %LVM) compared to manual delineation in the study by Amado et al. [8] and Hsu et al. [12] (8.6 ± 1.9 % LVM, *R* = 0.69 and 5.4 %, *R* = 0.96, respectively). Interobserver variability was analyzed in patients in a core lab setting and showed a lower bias and variability compared with previous studies by Flett et al. [9] and McAlindon et al. [10]. Thus, the expert delineation was used as reference in the patient population.

### Patient population

The EWA algorithm was applied in 124 patients from multi-center, multi-vendor studies with bias to expert delineation (−2 ± 6 %LVM) similar to the FACT algorithm by Hsu et al. [13] which was evaluated in 20 patients from a single center (3.8 %LVM, *R* = 0.95). Heiberg et al. [14] found a lower bias for the original weighted algorithm (0.3 ± 2.7 %LVM) in a two-vendor, single-center study of 40 patients. However, in the present study, the performance of the EWA algorithm was compared to the original weighted algorithm [14] and a higher bias and variability was found for the original weighted algorithm in the current multi-center, multi-vendor study than in the original study [14]. Similarly, variability was increased for the threshold by FWHM from minimum intensity and n-SD from remote in comparison to the validation against TTC by Amado et al. [8] and in contrast to the study by Hsu et al. [12] an underestimation was seen for the threshold of FWHM from remote. The changes in bias and variability seen in the current multi-center, multi-vendor patient study compared to previous validations in experimental studies [8, 12, 14] and single-center patient studies [13, 14] underlines the importance of using multi-center, multi-vendor patient data. Multi-center, multi-vendor patient data has a larger variability in image quality and thus the automatic algorithm is faced with a larger challenge which may not have been accounted for in the algorithm if designed and validated for single-center patient data or experimental data.

Additionally, infarct validation needs to be performed in both magnitude IR and PSIR images since both are used in clinical routine. Stirrat et al. [11] recently showed a significant bias of infarct size in paired magnitude IR and PSIR images for n-SD from remote and FWHM from minimum intensity. Based on their findings we compared infarct size in paired IR and PSIR images to expert delineation in 49 patients for the EWA algorithm, threshold methods of EM, 2, 3, and 5 SD, FWHM from minimum intensity and Otsu's threshold. There was a large bias between IR and PSIR images for the threshold of FWHM from minimum intensity and Otsu's threshold with underestimation in IR and overestimation in PSIR images whereas the bias for the EWA method was 1 ± 4 %LVM. Bias between IR and PSIR for 2, 3 and 5 SD was lower in this study than in the study by Stirrat et al. [11] (−3 %LVM, −4 %LVM and −5 %LVM, respectively) and is possibly explained by different definitions of remote region. In the present study the remote region was defined from the a priori maximal extent model for each culprit artery. In the study by Stirrat et al. [11] care was taken to manually define a large remote region, however, infarct size in controls without myocardial infarction was found as 14 %LVM by 2SD and 9 %LVM by 3SD instead of the theoretically defined 2 % and 0.1 %. The difficulty in defining a remote region representative of normal myocardium is also shown by high variability of 2SD in inter- and intra observer variability and test-retest repeatability found by both Flett et al. [9] and McAlindon et al. [10]. By using the EWA algorithm there is no need for manual definition of remote regions, and the EWA algorithm showed a lower variability and higher regional agreement than any other of the methods and a low bias and variability between IR and PSIR images.

### Limitations

The EWA algorithm was developed for quantification of myocardial infarction in both IR and PSIR LGE images and an intensity threshold was fitted using an EM algorithm. The EM algorithm was however implemented using two Gaussian distributions as opposed to the study by Hennemuth et al. [30] in which a Rayleigh-Gaussian mixture model was used. In the case where the myocardium is not properly nulled the distribution shifts from Rayleigh distribution to a more Gaussian distribution. This shift can also be caused by surface coil corrections applied at the scanner and the usage of both IR and PSIR images. In the current study the intensity histograms of the myocardial intensities were frequently perceived as being more representative of Gaussian distributions than of Rayleigh distributions and therefore the more generic approach of two Gaussian distributions was chosen.

The manual segmentation to which the EWA algorithm was tested in the patient data was initiated by a previously described computer algorithm [14] which may have a significant bias on the manual observer. That is why we in the present study include independent reference standard validation from the experimental data. That is also why we are describing the patient data as performance data, and not as validation, since there is no true reference standard for the patient data.

The EWA algorithm was applied in multi-center, multi-vendor patient data from clinical trials of first time STEMI and experimental studies of a single infarction and the EWA algorithm was developed for single vessel myocardial infarction. For multi-vessel myocardial infarction or multiple infarctions over time, the algorithm can, however, be used without the a priori information of culprit artery models. The algorithm would then not be able to apply the intensity correction and would need further validation for multi-vessel disease. For other types of myocardial fibrosis such as in the situation of hypertrophic cardiomyopathy and myocarditis both a priori information and post processing might need to be adjusted and would require additional validation for these groups of patients. However, the EWA algorithm was applied in the experimental data without the use of a priori information on culprit artery due to differences in anatomy and showed a low bias in IR and PSIR LGE images and ex-vivo high resolution T1-weighted images. The low bias found in T1-weighted images as well as in IR and PSIR LGE images shows the ability of the EWA algorithm to assess infarct size in a wide range of settings with a variety of different imaging strategies. The need for manual corrections was not assessed, however, considering the lower bias and higher regional agreement than for the original weighted algorithm less manual corrections would probably be needed. Especially for quantification in ex-vivo high resolution T1-weighted images, time will be saved by the limited amount of user input in comparison to definition of remote regions in all 0.5 mm slices covering the left ventricle.

## Conclusion

We have developed a new automatic algorithm, the EWA algorithm, for quantification of myocardial infarction in LGE images based on combining an intensity classification by Expectation Maximization (EM) with a pixel intensity weighting approach to account for partial volume effects. The EWA algorithm performed well for both magnitude IR and PSIR LGE images when validated in experimental studies against TTC and ex-vivo high resolution T1-weighted images, and when compared to expert delineations in multi-center, multi-vendor patient data. Thus, using EM and a weighted approach as with the EWA algorithm, may serve as a candidate for a clinical standard in quantifying myocardial infarction.
